# High-Fat Diet-Induced Fatty Liver Is Associated with Immunosuppressive Response during Sepsis in Mice

**DOI:** 10.1155/2021/5833857

**Published:** 2021-12-08

**Authors:** Fangzhao Wang, Zhongran Cen, Zhanguo Liu, Jianwei Gan, Xianglong Zhang, Qianru Cui, Shenhai Gong, Ping Chang, Peng Chen

**Affiliations:** ^1^Department of Intensive Care Unit, Zhujiang Hospital, Southern Medical University, Guangzhou, China; ^2^Department of Pathophysiology, Guangdong Provincial Key Laboratory of Proteomics, School of Basic Medical Sciences, Southern Medical University, Guangzhou, China

## Abstract

High-fat diet-induced fatty liver is an indolent and chronic disease accompanied by immune dysfunction and metabolic disturbances involving numerous biological pathways. This study investigated how this abnormal metabolic disorder influences sepsis in mice. Mice were fed with normal chow (NC) or high-fat diet (HFD), and palmitic acid (PA) was used to treat hepatocytes to mimic fat accumulation *in vitro*. Lipopolysaccharide (LPS) was used to induce sepsis and related immune responses. Mice fed on a high-fat diet displayed higher mortality and more severe liver damage but compromised immunoreaction. The supernatant from PA-treated primary hepatocytes markedly diminished the inflammatory cytokine expression of macrophages after LPS stimulation, which showed a state of immunosuppression. Metabolomics analysis indicated the level of many key metabolites with possible roles in immunoreaction was altered in the HFD and PA groups compared with corresponding controls; specifically, *β*-hydroxybutyric acid (BHB) showed an immunosuppressive effect on Raw264.7 cells during the LPS stimulation. Transcriptomic analysis suggested that several differential signaling pathways may be associated with the alteration of immune function between the NC and HFD groups, as well as in the *in vitro* model. Our study suggests that the consumption of HFD may alter the hepatic metabolic profile, and that certain metabolites may remold the immune system to immunosuppressive state in the context of sepsis.

## 1. Introduction

Sepsis represents the major cause of death in the intensive care unit (ICU) and induces long-term immune dysfunction symptoms. Normally, sepsis augments immune responses and boosts proinflammatory factor production upon initial infection, whereby overproduced cytokines directly damage organ cells leading to organ failure and death. However, during the progression of sepsis, immune reactions switch from an immune activation state to an immunocompromised state characterized by a failure to resist the primary infection and then progress to a more protracted disease due to recurrent lethal infections [1–3].

The application of immune-enhancing drugs in clinical trials has shown benefit indicating direct immunosuppressive therapy was effective [4]. Animal models of sepsis also confirm the hypothesis that boosting immunity improves survival [5].

The increasing worldwide prevalence of high-fat diet-induced obesity and fatty liver has become a serious public health burden in recent decades [6] and was reported to be strongly associated with immune dysfunction [7, 8] and to impair host immunity during bacterial infection [9]. Furthermore, energy imbalance and metabolite disturbance result in tissue stress and organ damage [10]. Recent studies of individuals with obesity reported they had physiological vulnerability, susceptibility, and a high risk of infection and mortality from sepsis [11, 12].

In this study, we used a rodent model of diet-induced obesity (DIO) to explore the detailed association between obesity-associated fatty liver abnormities and immune reactions in the context of sepsis. Using multiple omics analysis, we provide direct evidence that the intake of a high-fat diet caused a severe septicemia phenotype and markedly suppressed systemic immune inflammatory responses in mice. We further showed that this immunocompromised phenotype may be associated with hepatocyte metabolic disorder. Thus, our current study strengthens the hypothesis that a long-term high-fat diet is harmful to the immune balance and may be a risk factor for septic mortality.

## 2. Materials and Methods

### 2.1. Animals

All experimental procedures conformed to the Institutional Animal Care and Use Committee (IACUC) guidelines and were permitted by the related ethical regulations of the Laboratory Animal Ethics Committee of Southern Medical University. Four-week-old male C57BL/6 mice were maintained on a 12 h day/night cycle at 22 ± 2°C and fed with either NC (D12450J, 10 kcal% fat; Research Diets, NJ, USA) or HFD chow (D12492, 60 kcal% fat; Research Diets, NJ, USA) for 15 weeks. Body weight and food intake were supervised weekly. For the animal models of endotoxemia, mice were treated with LPS (SIGMA, L2630-100MG, 10 mg/kg) for 12 h. Then, tissue samples were collected under anesthesia.

### 2.2. Plasma Aminotransferase Assay

When blood samples were collected, plasma was separated after centrifugation at 12,000 × g for 10 min. Plasma aspartate aminotransferase (AST) was assessed using commercial kits (Nanjing Jiancheng Bioengineering Institute) according to the manufacturer's protocol.

### 2.3. Peritoneal Lavage Fluid (PLF) Collection and Flow Cytometry Detection

Under anesthesia, the abdominal cavity of mice in each group was intraperitoneally lavaged with 10 mL sterile Dulbecco's Phosphate Buffered Saline (DPBS, Gibco, C14190500BT) solution several times, and then, the PLF was collected in sterile centrifuge tubes, centrifuged at 1500 rpm for 5 min. The supernatant was discarded, and 1 mL sterile DPBS solution was added to resuspend the pellet. Neutrophils and macrophages were marked with fluorescein-labeled CD11b^+^/Ly-6G^+^ monoclonal antibody and CD11b^+^/F4/80^+^ monoclonal antibody (Biolegend, LY6-G, 127636; BD, F4/80, 564227; Biolegend, CD11b, 163702), respectively. After 30 min incubation at 4°C, they were washed twice and analyzed by flow cytometry (FACS).

### 2.4. Primary Hepatocyte Isolation and Culture

We utilized a method to isolate and culture primary hepatocytes as described previously [13]. Briefly, the procedure was conducted by two-step collagenase perfusion with collagenase type IV (Worthington, LS004188) and collagenase P from clostridium histolyticum (Roche, 11213857001), and the perfusate was retrograde and nonrecirculating. As the liver was gradually digested, we dissected it and then gently teased it into small pieces that were passed through a 100 *μ*m strainer (Falcon). After 3 min centrifugation at 800 rpm, cells were cultured in RPMI1640 medium (Gibco, C11875) with 10% fetal bovine serum (Thermo Fisher, 10099141C) and 1% penicillin/streptomycin (Gibco, 15140122) for 6-8 h for attachment on the plates in a 37°C incubator with 5% CO_2_. Notably, collagen I (BioCoat, 354236) was diluted with 30% absolute ethyl alcohol to 1.2% solution and then precoated on the plates followed by 30 min of ultraviolet radiation and 8 h of ventilation. The plates were washed three times with DPBS before use.

### 2.5. Albumin-Conjugated PA Solution Preparation

As reported [14], PA in 100% ethanol was complexed with 20% fatty acid-free bovine serum albumin (BSA) (VETEC, WXBC8945V) in DPBS at 50°C for 1 h to yield a final stock solution of 40 mM.

### 2.6. Triacylglycerol (TG) Measurements in the Liver and Hepatocytes In Vitro

When the primary hepatocytes had completely adhered, the cells were washed with DPBS three times and incubated in fresh medium containing 0.4 mM PA or BSA control. After 12 h of treatment, the washed cells received fresh medium for another 12 h; then, the supernatant was collected and stored at -80°C. To quantify the lipid accumulation, TG content was detected by commercial kits according to the manufacturer's instructions (Nanjing Jiancheng Bioengineering Institute, A110-1-1).

### 2.7. Cell Treatment

We seeded Raw264.7 cells at a confluence of 70-75% per well and treated them with LPS (1 *μ*g/ml) and hepatocyte supernatant for 24 h. *β*-hydroxybutyric acid (BHB, Sigma-Aldrich, 166898) was given 24 h with or without LPS to test its modulatory function on Raw264.7 cells.

### 2.8. Gene Expression Analysis

Total RNA was extracted from cells using TRIzol agent followed by a reverse transcription (RT) reagent kit (TOYOBO) to synthesize complementary DNA (cDNA). According to the manufacturer's instructions, the real-time PCR was conducted with a SYBR® Green Real-time PCR Master Mix (TOYOBO). All *^ΔΔ^C_t_* values were normalized to the house keeping gene *18*S. Primer sequences used for quantitative PCR are listed in Table 1.

### 2.9. Metabolomics Analysis

The metabolomics progress was performed based on a standard method as described previously [15, 16]. In short, standards were diluted at a concentration of 5.0 mg/ml. The Eppendorf epMotion Workstation (Eppendorf Inc., Germany) was operated when the samples were injected into 96-well plates mixed with methanol for 5 min and followed by centrifugation (4000 × g, 30 min). Samples were further diluted by the 50% methanol. Then, 135 *μ*l supernatant was transferred into a clean plate followed by LC-MS procedures. ACQUITY UPLC BEH 1.7 *μ*M VanGuard precolumn (2.1 × 5 mm) and ACQUITY UPLC BEH C18 1.7 *μ*M analytical column (2.1 × 100 mm) were used in this procedure. 0.1% formic acid water was used as mobile phase A, and 0.1% acetonitrile isopropanol was used as mobile phase B. The elution procedure was as follows: 0-1 min, 5% B; 1-5 min, 5-30% B; 5-9 min, 30-50% B; 9-11 min, 50-78% B; 11-13.5 min, 78-95% B; 13.5-14 min, 95-100% B; 14-16 min, 100% B; 16-16.1 min, 100-5% B; and 16.1-18 min, 5% B. The flow rate was 0.4 ml/min. For the mass spectrometer settlement, 1.5 (ESI+), 2.0 (ESI-) was used for capillary voltage, 150°C for source temperature, and 550°C for desolvation temperature. Data process and analysis were consistent with previous study [17].

### 2.10. Transcriptomic Analysis

The RNA from cells in PLF and Raw264.7 cells were isolated according to the instruction manual of the TRIzol reagent. The concentration and purity of RNA were evaluated by a NanoDrop Spectrophotometer (Thermo Fisher, USA) and Labchip GX Touch HT Nucleic Acid Analyzer (PerkinElmer, USA). High-quality RNA was used for cDNA library construction and sequencing (Wuhan Bioacme Biological Technologies Corporation, Wuhan, China). mRNA was then enriched by oligo (dT) beads. RNA sequencing libraries were generated using the KAPA Stranded RNA-Seq Kit for illumining with multiplexing primers. Then, sequencing was performed on an Illumina Nova sequencer.

We obtained high-quality clean reads with Fastp V0.20 to ensure the filtering quality of raw data. The position of the genome was compared according to the annotation file of the genome and reads, and the number of reads contained in each gene was counted to get the expression amount, and then, the differentially expressed genes were obtained by statistical tests.

Specifically, based on the negative binomial distribution as a model of RNA-Seq read number, we used the statistical software, DESeq2, to represent gene read number differences among biological individuals. DESeq provides statistical routines for determining differential expression in digital gene expression data using a model based on the negative binomial distribution. Genes with an adjusted *P* value < 0.05 found by DESeq were assigned as differentially expressed. The resulting *P* values were adjusted using Benjamini and Hochberg's approach for controlling the false discovery rate. Finally, functional annotation of differentially expressed genes was carried out to obtain GO and KEGG sets enriched by differentially expressed genes. Raw sequencing data have been uploaded to the BioProject database (https://www.ncbi.nlm.nih.gov/bioproject; accession number PRJNA739478), and all the raw sequencing data could be released upon reasonable request by contacting the authors.

### 2.11. Immunoblotting Analysis

RIPA lysis buffer (Beyotime) adding proteinase inhibitor and phosphatase inhibitors (Beyotime) were used to prepare Raw264.7 cell lysate. After centrifuging at 13000 × g for 20 minutes, the supernatant was collected for protein quantification (Pierce BCA protein Assay Kit). Equivalent proteins were separated by 12% SDS-PAGE; then, the proteins were transferred to PVDF membranes (Merck Millipore, IPVH00010) and incubated with antibodies as follows: p-AKT (1 : 1000, Cell Signaling Technology), AKT (1 : 1000, Cell Signaling Technology), and *β*-actin (1 : 1000, Cell Signaling Technology). Then, the secondary antibody (1 : 2000, Cell Signaling Technology) was used for further incubation. Ultrasensitive ECL Chemiluminescence Kit (Beyotime, P0018FS) was used to detect the bands. Blots were analyzed by ImageJ.

### 2.12. ELISA

The level of TNF-*α*, IL-6, and IL-10 was determined by commercial kit (NEOBIOSCIENCE, EMC102a.96, EMC004.96, and EMC005.96) according to the manufacturer's protocol.

### 2.13. Statistical Analysis

Data are expressed as the mean ± standard error of the mean (SEM). *P* values were calculated using the Student *t*-test, and values <0.05 indicated statistical significance.

## 3. Results

### 3.1. Mice on HFD Have Exacerbated Septic Symptoms

**Figure 1:**
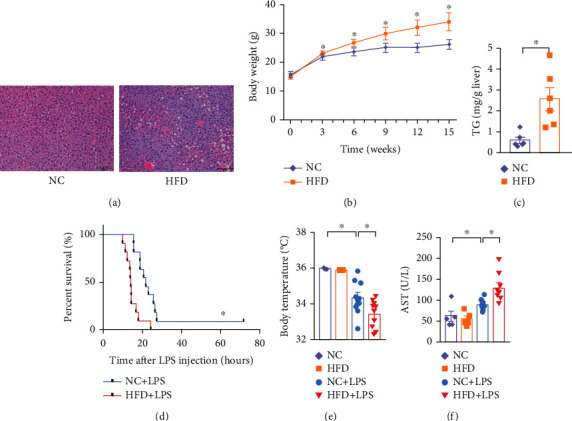
Mice on high-fat diet have aggravated septic symptoms. (a) Mice fed with normal chow (NC) or high-fat diet (HFD) for 15 weeks. Representative liver sections of hematoxylin and eosin (H&E) staining. (b) Changes in the body weight of mice fed NC (*n* = 36) or HFD (*n* = 37) for 15 weeks. (c) TG content in liver of mice fed NC (*n* = 6) or HFD (*n* = 6). (d) The survival rate of the NC group (*n* = 11) and the HFD group (*n* = 11) after LPS (10 mg/kg) intraperitoneal injection. (e) Body temperature in mice 12 h after LPS (10 mg/kg) intraperitoneal injection or control treatment. NC (*n* = 3), HFD (*n* = 3), NC+LPS (*n* = 10), and HFD+LPS (*n* = 10). (f) Plasma levels of AST (NC *n* = 5, HFD *n* = 5, NC+LPS *n* = 8, and HFD+LPS *n* = 8) were measured in NC and HFD groups injected with or without LPS (10 mg/kg). ^∗^*P* < 0.05 by two-tailed Student's *t-*test. Scale bars = 50 *μ*m.

We randomized mice into two groups fed with either a normal chow diet as a control (NC) or a high-fat diet (HFD). At the end of 15 weeks feeding, we observed obvious fat deposition in hepatocytes (Figure 1(a)). We also monitored mouse weight weekly (Figure 1(b)) and the triglyceride (TG) content in livers (Figure 1(c)). These data clearly confirmed HFD fed mice developed obesity and fatty liver.

To investigate the impact of HFD on the outcome of sepsis, we gave mice an intraperitoneal injection of lipopolysaccharide (LPS) and performed a 72 h survival test. HFD mice had higher mortality rates and shorter survival time compared with NC mice (Figure 1(d)). Furthermore, HFD mice subjected to LPS had a dramatically lower body temperature (Figure 1(e)) and increased plasma transaminase levels compared with the NC group (Figure 1(f)). Collectively, these observations demonstrate that HFD significantly augmented septic mortality and organ damage induced by LPS.

### 3.2. HFD Septic Mice Are in a State of Immune Suppression

**Figure 2:**
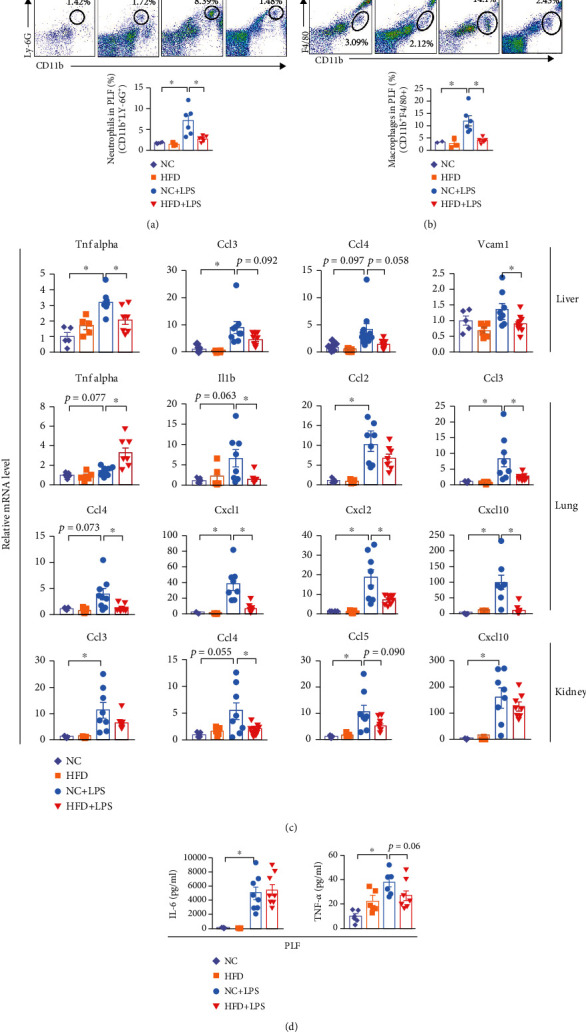
High-fat diet septic mice are in a state of immune dysregulation. (a) The number of neutrophils labeled with CD11b and LY6G in the peritoneal lavage fluid (PLF) of mice was determined by flow cytometry. NC (*n* = 2), HFD (*n* = 3), NC+LPS (*n* = 6), and HFD+LPS (*n* = 5). (b) The number of macrophages labeled with CD11b and F4/80 in PLF of mice was determined by flow cytometry. NC (*n* = 2), HFD (*n* = 3), NC+LPS (*n* = 6), and HFD+LPS (*n* = 5). (c) Gene expressions of inflammatory cytokines and chemokines (NC *n* = 5, HFD *n* = 5, NC+LPS *n* = 8, and HFD+LPS *n* = 8) analyzed by quantitative RT-PCR (relative to 18S). (d) The relative protein levels of TNF-*α* (NC *n* = 5, HFD *n* = 5, NC+LPS *n* = 7, and HFD+LPS *n* = 8) and IL-6 (NC *n* = 5, HFD *n* = 5, NC+LPS *n* = 8, and HFD+LPS *n* = 8) in PLF. ^∗^*P* < 0.05 by two-tailed Student's *t-*test.

Immune reactions have a key role in sepsis development. Next, we investigated whether HFD caused the dysregulation of the immune system. As measured by flow cytometry, the proportion of neutrophils and macrophages in the NC group was significantly increased in peritoneal lavage fluid (PLF) after LPS challenge (Figures 2(a) and 2(b)). However, when fed with a HFD, the ratio of neutrophils and macrophages in PLF was similar to the unchallenged state (Figures 2(a) and 2(b)). Consistent with these findings, the expression levels of most inflammatory mediators in the main organs (liver, lung, and kidney) were markedly decreased in HFD septic mice compared with NC septic mice (Figure 2(c)). Although the protein levels of interleukin-6 (IL-6) in PLF were comparable between NC and HFD septic mice, tumor necrosis factor alpha (TNF-*α*) levels were reduced comparatively after LPS challenge in HFD mice (Figure 2(d)). Taken together, these results suggest that the immune response of obese subjects to infection, characterized by impaired immune cell chemotaxis and dysregulated cytokine production, is lower than that of normal-weight subjects.

### 3.3. Palmitic Acid-Induced Hepatocyte Fat Accumulation Manifests as Impaired Immune Competence to LPS

**Figure 3:**
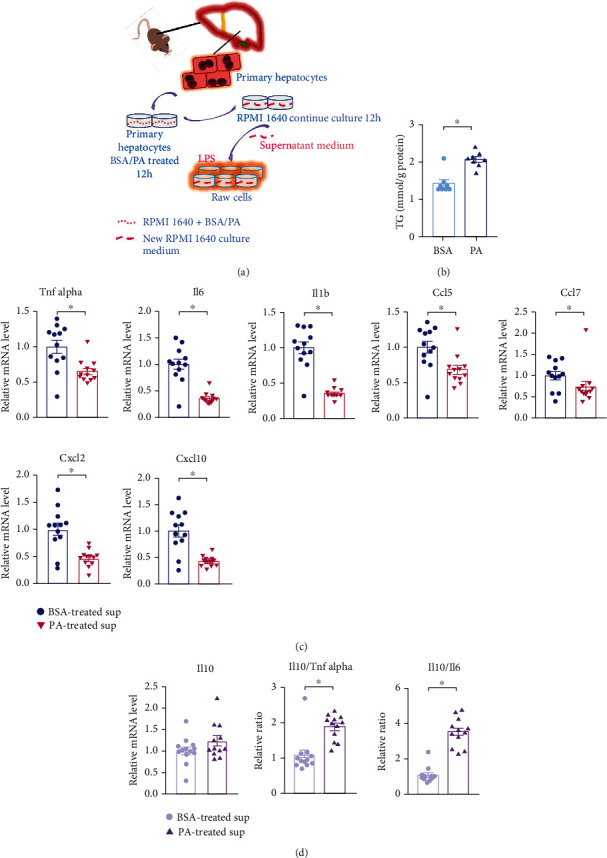
Palmitic acid-induced hepatocyte fat accumulation manifests as impaired immune competence to LPS challenge. (a) Murine primary hepatocytes were harvested from C57BL/6 mice and seeded in 12-well collagen I-coated plates and incubated in RPMI1640 medium. After attaching for 8 h, cells were washed three times with DPBS and stimulated with 0.4 mM PA or BSA control for 12 h. Then, the medium was replaced by the refresh medium to continue incubating for another 12 h. Finally, the supernatant was collected for further study. Raw264.7 cells were seeded at a confluence of 70-75% per well and treated with LPS (1 *μ*g/ml) and hepatocyte supernatant for 24 h. (b) After incubating with PA or BSA, the TG content of primary hepatocytes was measured. BSA (*n* = 8) and PA (*n* = 8). (c) Inflammatory cytokines and chemokines of Raw264.7 cells (group of BSA-treated Sup, *n* = 12 and group of PA-treated Sup, *n* = 12) in (a). (d) *Il-10* and immunosuppression markers *Il-10/Tnf alpha* and *Il-10/Il-6* mRNA levels of Raw264.7 cells, treated with BSA or PA-stimulated hepatocyte supernatant (group of BSA-treated Sup, *n* = 12 and group of PA-treated Sup, *n* = 12). Sup: supernatant. ^∗^*P* < 0.05 by two-tailed Student's *t*-test.

To further evaluate the role of fat on immune responses, we incubated primary hepatocytes with or without saturated fatty acids (palmitic acid, PA) to mimic the fatty liver model *in vitro*. After a 12 h incubation with PA or control agent (bovine serum albumin, BSA), the supernatant was replaced by fresh medium and cells were cultured for another 12 h. Then, the new supernatant was added to Raw264.7 cells, which were cotreated with LPS (Figure 3(a)). The accumulation of TG in hepatocytes after PA or BSA administration was confirmed (Figure 3(b)). Interestingly, compared with the control supernatants, administration of the supernatant from the PA group on Raw264.7 cells had decreased the mRNA levels of key inflammatory cytokines and chemokines including *Tnf alpha*, *Il-6*, *Il1b*, C–C chemokine ligand 5 (*Ccl5*), *Ccl7*, C-X-C motif chemokine ligand-2 (*Cxcl2*), and *Cxcl10* (Figure 3(c)), which were similar to the lower immune reactions observed in HFD-sepsis mice.

IL-10 has been classically known as an anti-inflammatory and immunosuppressive cytokine produced by activated immune cells [18]. As expected, the expression of *Il-10* displayed an increased trend upon LPS combined with PA supernatant stimulation (Figure 3(d)). A suppressed immune status during sepsis has been defined as sepsis-associated immunoparalysis, which has been related to fatal outcomes in patients [19–22]. Immunoparalysis in septic patients can be quantified by the ratio of Il-10 and Tnf or Il-6. Patients with higher Il-10/TNF ratio are considered as immune paralysis, which may result in reduced survival [23, 24]. As expected, we also observed increased immunosuppression markers after PA supernatant administration, including *Il-10/Tnf alpha* and *Il-10/Il-6* (Figure 3(d)). These data inspired us to speculate that fat accumulation in primary hepatocytes may generate key factors that cause immune suppression in macrophages in response to LPS infection.

### 3.4. HFD and PA Exposure Alters the Liver Metabolic Profile

**Figure 4:**
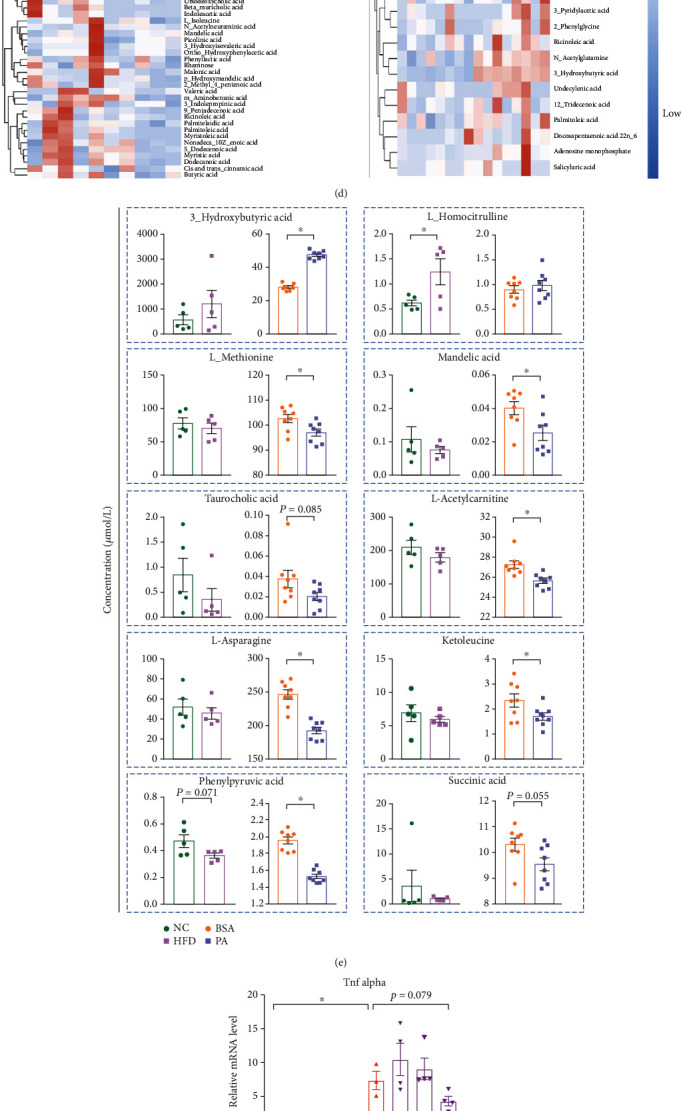
High-fat diet and palmitic acid exposure alter the liver metabolic profile. (a) Levels of TNF-*α*, IL-6, and IL-10 of BSA-Sup (*n* = 10) and PA-Sup (*n* = 8); (b) Scatter plots of principal component analysis (PCA, left panel) and orthogonal partial least squares discriminant analysis (OPLS-DA, right panel) in plasma metabolites of mice fed on normal chow (*n* = 5) or high-fat diet (*n* = 5). (c) Scatter plots of PCA (left panel) and OPLS-DA (right panel) in supernatant metabolites of primary hepatocytes treated with BSA (*n* = 8) or palmitic acid (*n* = 8). (d) Heat map analysis showing the changes of metabolites in mice fed on NC or HFD (left panel) and in the supernatants of primary hepatocytes challenged by BSA or PA. Fold changes of the two groups were >1.5 or <0.75. (e) Metabolites having the same abundance tendency *in vivo* (each group *n* = 5) and *in vitro* (each group *n* = 8). (f, g) mRNA levels of *Tnf alpha*, *Il-6*, *Il1b*, *Il-10*, *Tnf-α/Il-10* in Raw264.7 cells stimulated by BHB with or without LPS challenge (each group *n* = 3‐4). ^∗^*P* < 0.05 by two-tailed Student's *t*-test; ns: nonsignificant difference.

We next focus on the potential mechanism of PA-related supernatant mediating the immunosuppressive state of Raw264.7 cells. To completely understand the potential immune regulatory effects of hepatocyte-derived cytokines on Raw264.7 cell immune response, we first performed ELISA experiment to evaluate the proinflammatory (Tnf-*α* and IL-6) and anti-inflammatory cytokines (IL-10) from supernatant by BSA or PA administration. The data demonstrated that the content of the proinflammatory or anti-inflammatory cytokines showed no difference between BSA and PA treatment (Figure 4(a)**)**. These findings suggested that the immune response of Raw264.7 cells upon LPS challenge may be not regulated by the cytokines released from hepatocytes. We next to further determine whether the hepatocyte-derived metabolites which released by PA treatment or in plasma of HFD mice might lead to the immunosuppression of macrophages. We then conducted a metabolomics analysis of mouse plasma (NC vs. HFD, *in vivo*) and primary hepatocyte supernatant (BSA vs. PA, *in vitro*) to check whether the overall metabolic profile was shifted by fat deposition. Principal component analysis (PCA) and orthogonal projections with latent structure discrimination analysis (OPLS-DA) score plot analysis revealed a clear difference in metabolic profiles between the NC and HFD or BSA and PA groups (Figures 4(b) and 4(c)). We also used heat map visualization to identify the composition of metabolites in the plasma and cultured supernatants between treatment groups and their controls (Figure 4(d)). We then explored the metabolites that were altered under both *in vivo* and *in vitro* conditions. Compared with their controls, *β*-hydroxybutyric acid (BHB) was increased in the PA and HFD groups, whereas other compounds such as ketoleucine, mandelic acid, and phenylpyruvic acid were decreased (Figure 4(e)).

Recent discoveries have demonstrated the pleiotropic function of BHB, as a signaling metabolite mediating inflammation, oxidative stress, and gene expression [25]. Youm et al. proved that BHB could serve as an immune effector to inhibit the activation of NLRP3 inflammasome [26]. We thus designed to identify whether BHB plays a role in LPS-induced inflammatory response in vitro. We treated LPS-primed Raw264.7 cells with several concentrations of BHB. Interestingly, we found that BHB partially inhibited Raw264.7 cell response to LPS (Figure 4(f)). Meanwhile, BHB could increase *Il-10* expression in a dose -dependent manner. Most importantly, BHB increased *Il-10/Tnf alpha* ratio of macrophages in a dose-dependent manner, indicating that these BHB treated cells were in an immunosuppressed state (Figure 4(g)). In short, these experiments confirmed that BHB may play an immunomodulatory role in this model.

### 3.5. Transcriptomic Analysis Reveals Immune-Regulating Pathways Underlying HFD or PA Exposure

**Figure 5:**
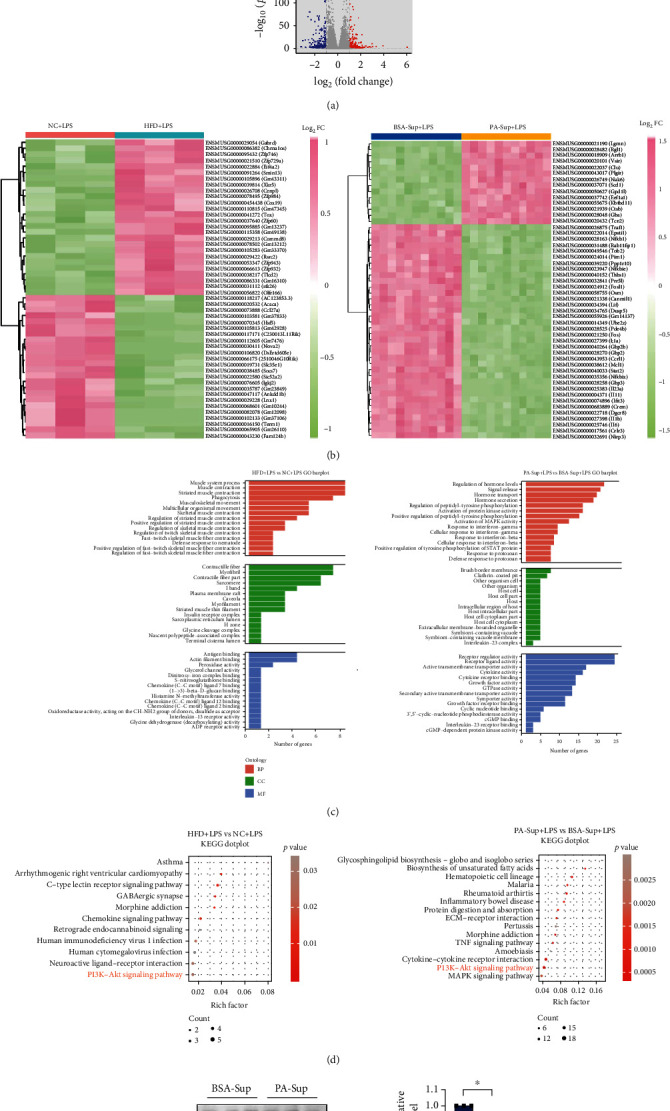
Transcriptomic analysis reveals septic immune regulatory pathways underlying high-fat diet or palmitic acid exposure. The RNA sample for transcriptomic procedure was derived from the PLF of NC/HFD mice and the Raw264.7 cells administrated with BSA/PA-related supernatant (BSA/PA-Sup) that are both under LPS treatment. (a) Volcano plot of HFD/NC+LPS (upper panel) and PA/BSA-Sup+LPS (lower panel) ratios (log_2_FC) versus -log_10_(*P* value) of gene expression. Red dots represent upregulated genes, while blue dots represent downregulated genes. (b) Differentially regulated genes of mice (RNA isolated from peritoneal lavage fluid of mice, *n* = 3) and cells (RNA isolated from Raw264.7 cells, *n* = 11) were identified by a hierarchical clustering heat map. (c) Gene Ontology (GO) enrichment analysis of differential genes between NC+LPS and HFD+LPS and BSA-Sup+LPS and PA-Sup+LPS groups. BP: biological process; CC: cellular component; MF: molecular function. (d) Differentially expressed genes analyzed by KEGG signaling pathway. (e) p-AKT/AKT levels. Raw264.7 cells were challenged with LPS (1 *μ*g/ml) and BSA-Sup or PA-Sup for 24 h. *n* = 3; ^∗^*P* < 0.05 by two-tailed Student's *t*-test.

Finally, we focused on immune cell responses by performing transcriptomic analysis of the inflammatory cells which were recruited to the abdominal cavity and Raw264.7 cells which were incubated LPS with PA-treated supernatant. Volcano plots highlight the transcripts that varied most between the NC and HFD or BSA and PA groups (Figure 5(a)). The top 50 genes with the highest variances of difference induced by PA or HFD were unsupervised clustered and represented in Figure 5(b). After screening, among the 50 genes with expression differences between the NC and HFD groups (*P* < 0.05), 26 genes were markedly higher and 24 were markedly lower in the HFD exposure group. Besides, among the 50 genes with expression differences between the BSA and PA groups (*P* < 0.05), 14 genes were markedly higher and 36 were markedly lower in the PA exposure group. A list of differentially enriched genes was produced according to Gene Ontology (GO) categories, including biological process (BP), cellular component (CC), and molecular function (MF), which showed divergent functional specialization in inflammatory cells in the PLF and Raw264.7 cells (Figure 5(c)). Using KEGG pathway analysis, we found the PI3K-AKT signaling pathway was involved both in the NC/HFD and BSA/PA groups (Figure 5(d)), indicating it might mediate immune regulation by fatty acids during sepsis development. Previous studies have demonstrated that the phosphorylation level of AKT was downregulated in HFD-fed mice [27, 28]. According to this, here we aim to further determine the level of PI3K/AKT signaling in lipid overload model with LPS administration. Indeed, compared with the BSA-Sup+LPS group, the level of p-AKT was diminished in PA-Sup cotreatment with LPS (Figure 5(e)), which was partly consistent with previous evidence [29], and this data further suggested PI3K/AKT pathway may be involved in the immune-modulation during fat overload challenge.

## 4. Discussion

In this study, the data showed that mice fed with HFD had higher mortality and severe organ damage compared to the control diet. Unexpectedly, the systemic and organ inflammation was reduced in HFD mice after LPS challenge, which was consistent with functional impairment including a large decrease in cytokine secretion in immunosuppressed septic patients [30]. Some investigators consider the idea of sepsis patients entering an immunosuppressive state is controversial because immune blocking therapy is still effective to some extent [31]. Recent studies have improved our understanding of sepsis immunopathology, including its initiation by complex cytokine-mediated hyperinflammation with the concomitant occurrence of profound immunosuppressive signs and deteriorating consequences [1, 3, 32, 33]. Furthermore, a clinical study revealed that the etiologies of patients who die in the ICU are in accordance with the characteristics of immune paralysis [30].

Here, we supposed that there is an immunosuppressive response in lipid overload environment upon septic challenge; we first treated primary hepatocytes with palmitic acid (PA) to mimic the lipid overload model *in vitro*. Considering the reagent interference, after 12 h treatment with BSA or PA, the culture medium of hepatocytes was replaced by the fresh medium for another 12 h incubation. Then, we collected the supernatant of two groups to further stimulate Raw264.7 cells with LPS for 24 h, respectively. This step is designed to further confirm whether the hepatocyte-derived metabolites produced in a fat overload environment have an impact on the immune response of macrophages. Indeed, Raw264.7 cells received PA-related supernatant exhibited an immunosuppressed status under LPS stimulation, with lower expression level of several proinflammatory cytokines and the higher ratio of *Il-10/Tnf-α* and *Il-10/Il-6* (Figures 3(c) and 3(d)).

Scientific achievements have highlighted the central role of metabolic failure, epigenetic programming events, transcriptional profiles, and other cellular alterations in the hyperinflammatory state and immune-tolerant state of sepsis development [34]. Moreover, differences in the metabolic profiles of immune cells during sepsis have been reported [35]. Globally, increasing numbers of people are obese with energy imbalance and caloric overconsumption, which causes metabolic disturbances, organ dysfunction, and even tissue stress [30]. In a study of >1000 sepsis patients, the investigators examined the plasma metabolomes (and proteomes) of patients who ultimately died as well as patients who survived. The analysis indicated that fatty acid transport and *β*-oxidation, gluconeogenesis, and the citric acid cycle were different in these two groups [36]. Furthermore, the role of macrophage metabolism has been studied recently. Intriguingly, the maintenance of proinflammatory (M1) macrophages relies on glucose metabolism and lactate production, whereas anti-inflammatory (M2) macrophages depend on Oxphos [35, 37] and *β*-oxidation through fatty acid uptake [38, 39]. These conclusions clearly pointed out that immune cell function closely relies on the metabolic microenvironment.

We next focused on which molecular probably mediated this immunosuppressive status in our model. Metabolomics program was then performed to further identify which potential metabolites had immunosuppressive effects, and our additional data indicated macrophages treated with *β*-hydroxybutyric acid (BHB) presented an immunosuppressed status upon LPS stimulation (Figures 4(f) and 4(g)). Recent studies reported that BHB blocked the activation of NLR family pyrin domain-containing protein 3- (NLRP3-) associated inflammation and alleviated NLRP3 inflammasome-mediated disease [26, 40]. In addition, BHB inhibited innate immune responses at certain concentrations [26]. Another study demonstrated that the loss of an anti-inflammatory metabolite such as BHB may be a contributing factor in patients with alcohol hepatitis [41]. In addition, this study reported that BHB supplementation increased Il-10 levels and the M2 phenotypic macrophages to promote its immune modulating function [41]. Besides, BHB is an important energy source for the human body, and Du et al. demonstrated that excessive presence of free fatty acids and BHB was associated with severe fatty liver in cows and was harmful to the physiological metabolism [42]. And BHB could modulate the immune response in dairy cows by negatively affecting the bacterial phagocytic function of neutrophils [43].

Sepsis is a biphasic disease that starts with an initial inflammatory phase characterized by immune hyperactivation followed by a prolonged immunosuppression phase [3]. Several clinical trials demonstrated that drugs targeting the inflammatory phase of sepsis failed to improve the patient's condition [44], probably because inflammation is a necessary process in sepsis. In our study, during the development of fatty liver disease, we speculated that excessive BHB may markedly suppress or even impair the immune response, suggesting a weak immune response is insufficient to fight infection [45]. There is substantial evidence that the most deleterious effects of sepsis are caused by damage to the host response [46, 47], indicating the host immune response may be a treatment target. Although how HFD metabolites such as BHB influence the capacity of immune cells is unclear, our findings indicate it is important to investigate how our diet adjusts the sepsis immune tolerance state.

In addition, we investigated whether the inflammatory ability of immune cells was changed by this diet pattern or fatty acid environment. Transcriptomic analyses were performed on RNA isolated from peritoneal lavage fluid in NC/HFD-sepsis mice and Raw264.7 cells *in vitro*. Gene Ontology (GO) analyses indicated certain pathways were involved, including the activation of MAPK activity, which was reported to induce IL-10 production in regulatory T cells, which mediate immune paralysis in cancer [48]. Furthermore, STAT signaling regulated the expression of immunosuppressive proteins including PD-L1 [49].

We also found that the PI3K-AKT signaling pathway was enriched in the KEGG analysis of immune cells from mice and macrophage cell lines. Phosphatidylinositide 3 kinases (PI3Ks) and their downstream mediator, AKT, regulate many important cellular processes, including cell growth, survival, differentiation, and proliferation [50, 51], and this signaling pathway is also aberrantly activated in several human cancers [52, 53]. Previous study has demonstrated that the exposure of ketone body could downregulate the PI3K/PKB signaling cascade to promote the insulin resistance [29]. Particularly, downregulation of AKT was necessary for the suppressive capacity of Treg cells *in vivo* and *in vitro* [54–56]. However, when using an AKT activator, the immunosuppressive function could be abolished in PD-1 deficient Treg cell [57]. In the present study, KEGG pathway analysis showed the differential expression genes were enriched in the PI3K-AKT pathway, partly revealing the immunosuppressive response may be associated with this pathway (Figure 5(d)). In fact, we found the phosphorylation of p-AKT during LPS stimulation in PA-related supernatant group was also diminished (Figure 5(e)), which may explain that the decreased activation of PI3K/AKT signaling probably modulates the immunosuppressive function of Raw264.7 cells under PA-Sup+LPS treatment (Figure 3(c)).

In the present study, models of HFD coupled with metabolic factors have helped us to understand the progression of sepsis in fatty liver individuals. These findings prompt future studies to determine how and why HFD promotes sepsis in patients, which is vital to improving the quality of life for these patients. In the future, the investigation of sepsis-related shifts in the immune and metabolic profile might help identify novel therapeutic targets for sepsis.

## Figures and Tables

**Table 1:** Primers for qPCR.

	Left primer (5′-3′)	Right primer (5′-3′)
18 s	CGATCCGAGGGCCTCACTA	AGTCCCTGCCCTTTGTACACA
Tnf alpha	CCACCACGCTCTTCTGTCTAC	AGGGTCTGGGCCATAGAACT
Il-6	TGATGCACTTGCAGAAAACA	ACCAGAGGAAATTTTCAATAGGC
Il1b	GGTCAAAGGTTTGGAAGCAG	TGTGAAATGCCACCTTTTGA
Ccl2	CCTGCTGTTCACAGTTGCC	ATTGGGATCATCTTGCTGGT
Ccl3	ACCATGACACTCTGCAACCA	GTGGAATCTTCCGGCTGTAG
Ccl4	CATGAAGCTCTGCGTGTCTG	GAAACAGCAGGAAGTGGGAG
Ccl5	GTGCCCACGTCAAGGAGTAT	CCACTTCTTCTCTGGGTTGG
Ccl7	CTGCTTTCAGCATCCAAGTG	TTCCTCTTGGGGATCTTTTG
Vcam1	TCCGCTACCATCACCGTGTAT	TAGCCAGCACCGTGAATGTG
Cxcl1	ACCCAAACCGAAGTCATAGC	TCTCCGTTACTTGGGGACAC
Cxcl2	CGGTCAAAAAGTTTGCCTTG	TCCAGGTCAGTTAGCCTTGC
Cxcl10	CTCATCCTGCTGGGTCTGAG	CCTATGGCCCTCATTCTCAC
Il-10	GCTGGACAACATACTGCTAACC	ATTTCCGATAAGGCTTGGCAA

## Data Availability

Raw sequencing data have been uploaded to the BioProject database (http://www.ncbi.nlm.nih.gov/bioproject;accession number PRJNA739478). All data used to support the findings of this study are available from the corresponding author upon request.
